# *Pseudomonas aeruginosa* flagellum is critical for invasion, cutaneous persistence and induction of inflammatory response of skin epidermis

**DOI:** 10.1080/21505594.2018.1480830

**Published:** 2018-08-02

**Authors:** Magali Garcia, Eric Morello, Julien Garnier, Christine Barrault, Martine Garnier, Christophe Burucoa, Jean-Claude Lecron, Mustapha Si-Tahar, François-Xavier Bernard, Charles Bodet

**Affiliations:** a Laboratoire Inflammation Tissus Epithéliaux et Cytokines EA 4331, Université de Poitiers , Poitiers, France; b Laboratoire de Virologie et Mycobactériologie, CHU de Poitiers , Poitiers, France; c Centre d'Etude des Pathologies Respiratoires, INSERM UMR 1100, Université de Tours , Tours, France; d Bioalternatives , Gençay, France; e Laboratoire de Bactériologie et Hygiène, CHU de Poitiers , Poitiers, France; f Laboratoire d’Immunologie et Inflammation, CHU de Poitiers , Poitiers, France

**Keywords:** Bacterial pathogenesis, human keratinocytes, type 2 secretion system, type 3 secretion system, cytokines, chemokines, antimicrobial peptides, flagellin

## Abstract

*Pseudomonas aeruginosa*, an opportunistic pathogen involved in skin and lung diseases, possesses numerous virulence factors, including type 2 and 3 secretion systems (T2SS and T3SS) and its flagellum, whose functions remain poorly known during cutaneous infection. Using isogenic mutants deleted from genes encoding each or all of these three virulence factors, we investigated their role in induction of inflammatory response and in tissue invasiveness in human primary keratinocytes and reconstructed epidermis. Our results showed that flagellum, but not T2SS and T3SS, is involved in induction of a large panel of cytokine, chemokine, and antimicrobial peptide (AMP) mRNA in the infected keratinocytes. Chemokine secretion and AMP tissular production were also dependent on the presence of the bacterial flagellum. This pro-inflammatory effect was significantly reduced in keratinocytes infected in presence of anti-toll-like receptor 5 (TLR5) neutralizing antibody. Bacterial invasion of human epidermis and persistence in a mouse model of sub-cutaneous infection were dependent on the *P. aeruginosa* flagellum. We demonstrated that flagellum constitutes the main virulence factor of *P. aeruginosa* involved not only in early induction of the epidermis inflammatory response but also in bacterial invasion and cutaneous persistence. *P. aeruginosa* is mainly sensed by TLR5 during the early innate immune response of human primary keratinocytes.

## Introduction

Skin is a primary interface between the body and the environment providing a first line of defense against a broad array of pathogens and traumas [1]. Epidermis, a multilayered stratified epithelium mainly constituted of keratinocytes, is the most superficial layer of the skin [1,2]. Keratinocytes act as an immunological barrier expressing various toll-like receptors (TLRs) and nod-like receptors for recognition of pathogen-associated molecular patterns (PAMPs) [3,4] and secreting different kind of antimicrobial peptides (AMPs), cytokines and chemokines [5]. In this way, keratinocytes play a major role in innate immunity and in protection against infectious pathogens [2].

*Pseudomonas aeruginosa* is an opportunistic microorganism representing a major cause of health-care associated infections and pulmonary infections of cystic fibrosis patients [6]. This ubiquitous Gram-negative bacterium belongs to the transient skin microflora when the epithelial barrier function is compromised (*e.g*. wound, burn, ulcer…] or in moist regions of the skin of just 2% of non-hospitalized healthy persons [7]. *P. aeruginosa* is involved in acute cutaneous infections in which the skin is a primary focus, usually occurring in patients with a local contributive factor [8] or in which cutaneous lesions are a consequence of a bacteremia, mostly in immunocompromised patients [9]. In addition, this bacterium can be involved in infection of chronic wounds [10] and has been suggested to play a role in the pathophysiology of atopic dermatitis [11].

*P. aeruginosa* possesses many virulence factors such as pili, lipopolysaccharide (LPS), extracellular enzymes, exotoxins, a flagellum or five types of secretion systems (TSSs). This bacterium has a single polar flagellum that is crucial for motility, chemotaxis and adhesion [12,13]. The external part of the bacterial flagellum is composed of a short proximal hook and a long helical filament made up of flagellin (Fla) sub-units [14]. Seven different TSSs have been described in Gram-negative bacteria: type I to VI and IX (T1SS to T9SS), which are responsible for the export of ‘effector’ proteins into the extracellular milieu or into target host cells [15,16] . T1SS, T2SS, T3SS, T5SS and T6SS can be found in *P. aeruginosa*. Among them, T2SS and T3SS are considered as the major virulence factors of the *P. aeruginosa* involved in host-pathogen interactions. T2SS is composed of multiprotein secretions leading to the secretion of exotoxin A, LasA and LasB proteases, type IV protease, lipolytic enzymes and phospholipase H [17]. T3SS of *P. aeruginosa* contributes the direct injection of exotoxins U, S, T, and Y into mammalian cells. All together, T2SS and T3SS play a major role in acute infections, thereby enhancing disease severity [18–22].

The innate immune system is able to detect *P. aeruginosa* products such as LPS, flagellin and CpG DNA *via* TLR4, TLR5 and TLR9 respectively [23–25] leading to the induction of large amounts of pro-inflammatory cytokines such as interleukin (IL)-6, granulocyte-colony stimulating factor (G-CSF), tumor necrosis factor (TNF)α [24,26] during lung infections in mouse model. Moreover, studies have suggested the role of TLR5 in induction of an early innate response and of T2SS and T3SS, in death, due to *Pseudomonas* infection in mouse lung infection model [18,26,27].

As very little is known about the impact of the virulence factors of *P. aeruginosa* during cutaneous infection, the aim of this study was to investigate the role of T2SS, T3SS and the flagellum, in the induction of the inflammatory response of human primary epidermal keratinocytes and in the bacterial invasion potential of human epidermis and bacterial persistence in a subcutaneous skin infection mouse model. For this purpose, infections were effectuated using a wild-type *P. aeruginosa* strain (PAK), and isogenic mutants designed from this wild-type strain deleted from genes encoding the subunit of the flagellum: the flagellin (*ΔfliC*), T2SS (*ΔxcpQ*), T3SS (*ΔpscF*) or all of them (*ΔfliCΔxcpQΔpscF*). We investigated the expression of various chemokines, pro-inflammatory cytokines and antimicrobial peptides known to be produced by keratinocytes in infectious contexts and exerting leucocyte chemotactic activities, orchestrating skin inflammation through cell activation or having antimicrobial effects.

## Results

As a first step, the inflammatory response induced by wild-type *P. aeruginosa* strain (PAK), and isogenic mutants (Fla^−^, T2SS^−^, T3SS^−^ and Fla^−^/T2SS^−^/T3SS^−^) was characterized during monolayered primary human keratinocyte infection.

### Cytokine and AMP mRNA expression in response to keratinocyte infection by *P. aeruginosa* strains

**Figure 1.**
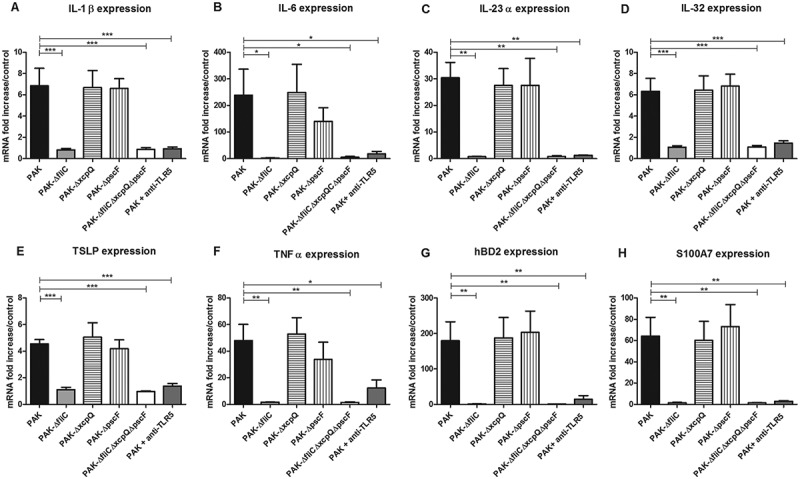
Cytokine and AMP mRNA expression in response to keratinocyte infection by each kind of bacterial strain. IL-1β (A), IL-6 (B), IL-23α (C), IL-32 (D), TSLP (E), TNFα (F), hBD2 (G) and S100A7 (H) mRNA expression by keratinocytes infected for 6 h with wild-type PAK strain, PAK ∆*fliC* (Fla^−^), PAK∆*xcpQ* (T2SS*^−^*), PAK∆*pscF* (T3SS*^−^*), PAK ∆*fliC*∆*xcpQ*∆*pscF* (Fla^−^ */*T2SS*^−^*/T3SS*^−^*) strains or wild-type PAK strain in presence of the anti-TLR5 monoclonal antibody. mRNA expression levels are expressed as the fold increase above unstimulated cultures. Data are represented as mean + SEM of five independent experiments. *p < 0.05, **p < 0.01 and *** p < 0.001.

Keratinocyte infection by *P. aeruginosa* wild-type strain (PAK) resulted in an induction of a large panel of pro-inflammatory cytokines including IL-1β, IL-6, IL-23α, IL-32, thymic stromal lymphopoietin (TSLP) and TNFα at 6 h post-infection (Figure 1(A–F)). *P. aeruginosa* wild-type strain also induced expression of AMP mRNA including human beta-defensin (hBD)2, S100 calcium-binding protein (S100)A7 after 6 h of infection (Figure 1(G,H)), as did S100A8, S100A9 and cathelicidin LL37 (supplementary figure 1) but not hBD3 and ribonuclease (RNase)7 mRNA expression (data not shown).

In comparison, keratinocyte infection with ∆*fliC* (Fla^−^) or triple mutant strains (*ΔfliCΔxcpQΔpscF*; Fla^−^/T2SS^−^/T3SS^−^) or with wild-type strain in presence of TLR5 blocking antibody showed significantly reduced induction of these cytokine and AMP mRNA expression (Figure 1). Infections with T2SS or T3SS mutant strains led to quite similar levels of cytokine and AMP mRNA expression by keratinocytes as compared to wild-type strain. At a later time of infection (16 h), reduced expression of IL-23α, TSLP, hBD2 and S100A7 was observed following keratinocyte infection with T3SS mutant strain compared to wild-type strain (supplementary figure 2).

### Chemokine mRNA expression and secretion in response to keratinocyte infection with *P. aeruginosa* strains

**Figure 2.**
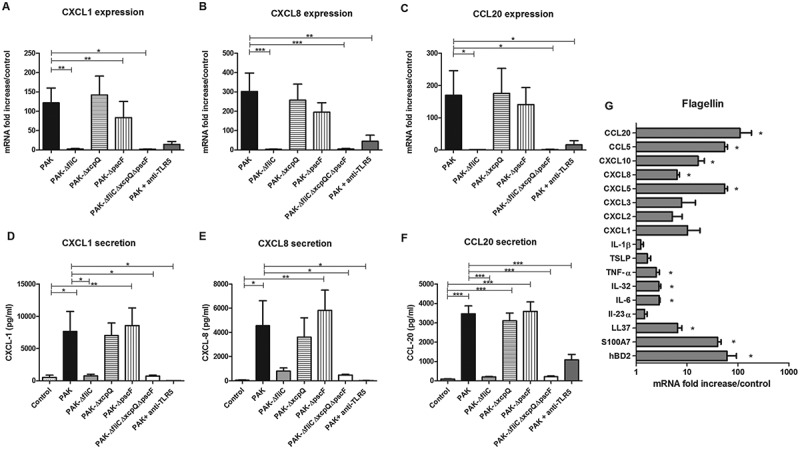
Chemokine mRNA expression and secretion in response to keratinocyte infection by each kind of bacterial strain and inflammatory response to flagellin stimulation. CXCL1, CXCL8, CCL20 mRNA expression (A to C) and secretion (D to F) by keratinocytes infected for 6 h with wild-type PAK strain, PAK ∆*fliC* (Fla^−^), PAK ∆*xcpQ* (T2SS*^−^*), PAK ∆*pscF* (T3SS*^−^*), PAK ∆*fliC*∆*xcpQ*∆*pscF* (Fla^−^ */*T2SS*^−^*/T3SS*^−^*) strains or wild-type PAK strain in presence of the anti-TLR5 monoclonal antibody. mRNA expression levels are expressed as the fold increase above unstimulated cultures. Protein concentrations (pg/mL) were measured in culture supernatants by ELISA assays. G: Cytokine, chemokine and AMP mRNA induction after 6 h of keratinocyte stimulation with purified flagellin from *P. aeruginosa* (1 µg/mL). Data are represented as mean + SEM of five independent experiments. *p < 0.05, **p < 0.01 and *** p < 0.001.

*P. aeruginosa* wild-type strain led to marked induction of CXCL1, CXCL2, CXCL5, CXCL8, CXCL10, CCL5 and CCL20 mRNA expression after 6 h of keratinocyte infection (Figure 2(A–C) and supplementary figure 3) as well as CXCL1, CXCL8 and CCL20 secretion quantified by ELISA (Figure 2(D–K)). Keratinocyte infection with ∆*fliC* (Fla^−^) or triple mutant strains (*ΔfliCΔxcpQΔpscF*; Fla^−^/T2SS^−^/T3SS^−^) or with wild-type strain in presence of TLR5 blocking antibody resulted in significantly reduced mRNA expression and secreted levels of chemokines. T2SS or T3SS mutant strains induced a chemokine response similar to the wild-type strain (Figure 2(D–K)). Chemokine expression analysis revealed that T3SS mutant induced lower mRNA levels of CXCL1, CXCL2, CXCL5, CXCL10 and CCL20 than the wild-type strain at 16 h post-infection (supplementary figure 4). In presence of TLR2 or TLR4 blocking antibodies, no modulation of the inflammatory response induced by wild-type strain was observed (data not shown). The TLR5-dependent inflammatory response induced by *P. aeruginosa* in keratinocytes was confirmed using another bacterial strain (ATCC 27,853) (supplementary figure 5). Ultrapure *P. aeruginosa* flagellin induced a profile of inflammatory mediator expression similar to the wild-type PAK strain but at a lower level (Figure 2(G) and supplementary figure 6). The inflammatory response induced by *P. aeruginosa* flagellin is significantly reduced in presence of TLR5 blocking antibody (supplementary figure 7).

As a second step, evaluation of the inflammatory response induced by the different bacterial strains was carried out on a three-dimensional model of reconstructed human epidermis (RHE) mimicking the multilayered epithelial structure of the epidermis (*stratum basale, stratum spinosum, stratum granulosum*, and *stratum corneum*).

### Cytokine and AMP mRNA expression of RHE in response to *P. aeruginosa infection*

**Figure 3.**
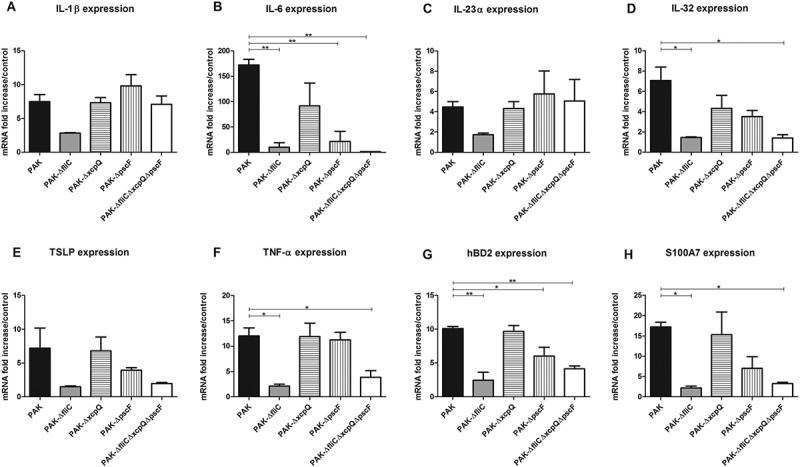
Cytokine and AMP mRNA expression in response to RHE infection by each kind of bacterial strain. IL-1β (A), IL-6 (B), IL-23α (C), IL-32 (D), TSLP (E), TNFα (F), hBD2 (G), S100A7 (H) mRNA expression in fold increase by RHE infected for 16 h with wild-type PAK strain, PAK ∆*fliC* (Fla^−^), PAK ∆*xcpQ* (T2SS*^−^*), PAK ∆*pscF* (T3SS*^−^*), PAK ∆*fliC*∆*xcpQ*∆*pscF* (Fla^−^ */*T2SS*^−^*/T3SS*^−^*) strains. mRNA expression levels are expressed as the fold increase above unstimulated cultures. Data are represented as mean + SEM of three independent experiments. *p < 0.05 and **p < 0.01.

An increase of IL-1β, IL-6, IL-23α, IL-32, TSLP, TNFα, hBD2 and S100A7 mRNA expression by RHE stimulated with wild-type strain was observed after 16 h of infection (Figure 3(A–H)). In agreement with the data on infected keratinocytes, RHE infected with ∆*fliC* or triple mutant strains showed significantly reduced induction of IL-6, IL-32, TNFα, hBD2 and S100A7 mRNA expression. While RHE infection with T2SS mutant had no effect on cytokine and AMP expression levels, infection with T3SS mutant strain resulted in significantly reduced expression of IL-6 and hBD2.

### Chemokine mRNA expression and secretion in response to reconstructed epidermis infection with *P. aeruginosa*

**Figure 4.**
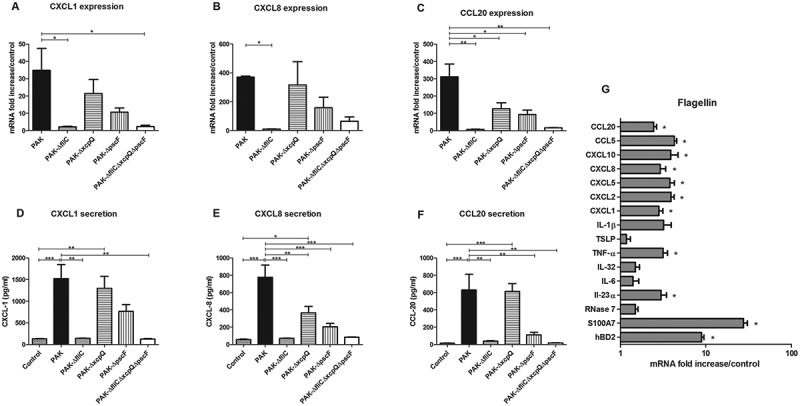
Chemokine mRNA expression in response to RHE infection by each kind of bacterial strain and inflammatory response to flagellin stimulation. CXCL1, CXCL8, CCL20 mRNA expression (A to C) and secretion (D to F) by RHE infected with wild-type PAK strain, PAK ∆*fliC* (Fla^−^), PAK ∆*xcpQ* (T2SS*^−^*), PAK ∆*pscF* (T3SS*^−^*) or PAK ∆*fliC*∆*xcpQ*∆*pscF* (Fla^−^ */*T2SS*^−^*/T3SS*^−^*) strains. G: Cytokine, chemokine and AMP mRNA induction after 16 h of RHE stimulation with purified flagellin from *P. aeruginosa* (1µg/mL) in culture medium. mRNA expression levels are expressed as the fold increase above unstimulated cultures. Protein concentrations (pg/mL) were measured in culture supernatants by ELISA assays. Data are represented as mean + SEM of five independent experiments. *p < 0.05, **p < 0.01 and *** p < 0.001.

CXCL1, CXCL2, CXCL8, CXCL10 and CCL20 mRNA expression by RHE infected with wild-type strain markedly increased at 16 h post-infection (Figure 4(A–C) and supplementary figure 8). On the other hand, RHE infection with ∆*fliC* or triple mutant strains resulted in reduced mRNA expression of these chemokines and secretion of CXCL1, CXCL8 and CCL20 (Figure 4(D–F)). Diminished CCL20 mRNA expression following infection with T2SS and T3SS mutant strains was also observed. RHE infection with T3SS mutant strain led to reduced CXCL8 and CCL20 secretion levels, whereas RHE infection with T2SS mutant strain resulted only in decreased secretion of CXCL8 (Figure 4(E–F)). CXCL5 and CCL5 mRNA expression were respectively not and weakly induced during RHE infection with wild-type strain and indistinctly modulated by the different mutants (data not shown). Ultrapure *P. aeruginosa* flagellin deposited on the upper layer of RHE did not result in induction of inflammatory mediator expression (data not shown), while flagellin added to RHE culture medium (in contact with the basal layer) induced IL-23α, TNFα, CXCL1, CXCL2, CXCL5, CXCL8, CXCL10, CCL5, CCL20, hBD2 and S100A7 mRNA expression (Figure 4(G)).

### Immunohistochemistry analysis of AMP expression by RHE infected with *P. aeruginosa*

**Figure 5.**
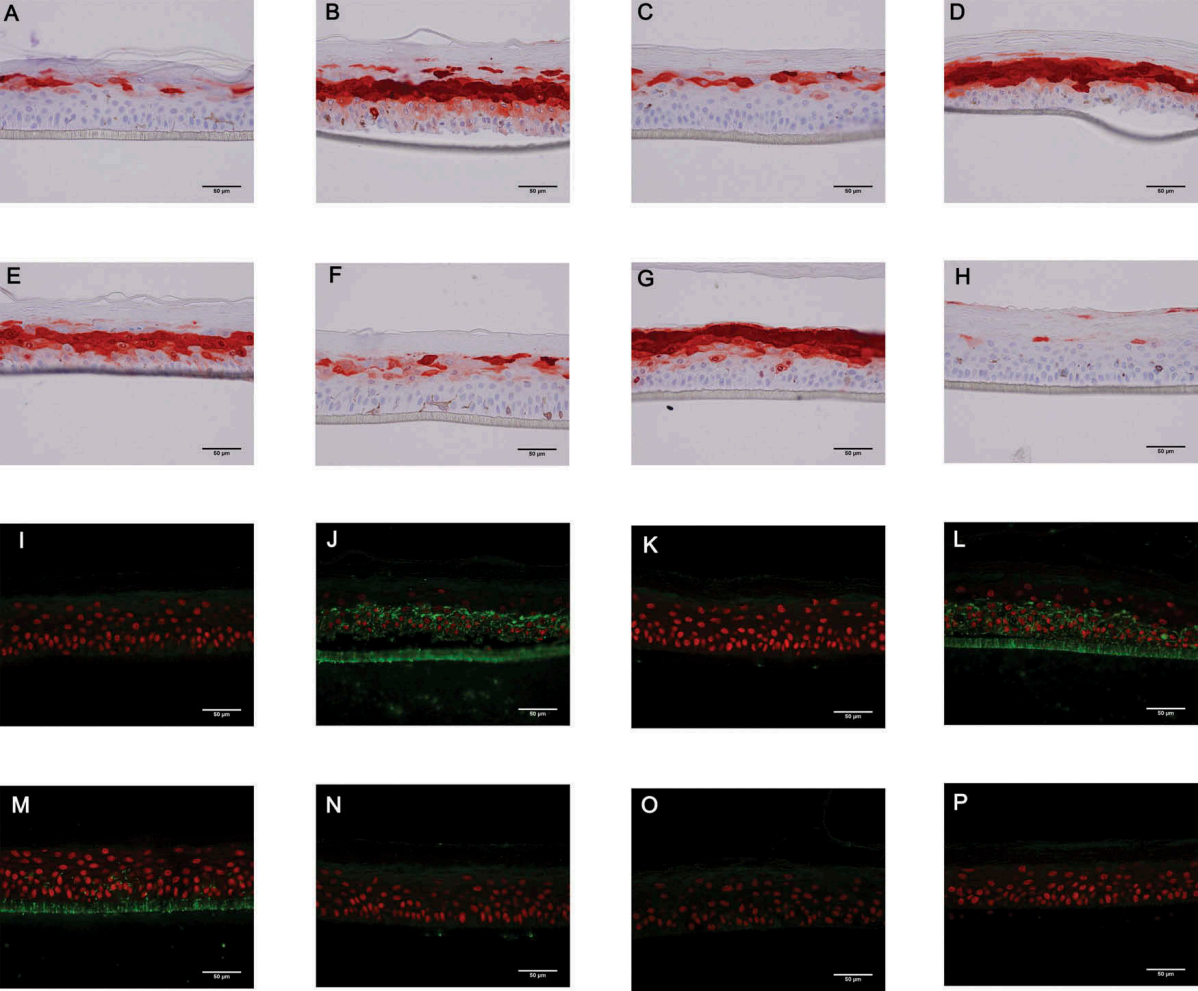
A to H: Level of S100A7 tissue production (red) assessed by immunohistochemistry on RHE infected with the different PAK strains of *P. aeruginosa* for 24 h. Nuclei were counter-stained using a hematoxylin solution (blue). **A**: control (sterile PBS), **B**: wild-type PAK strain, **C**: PAK ∆*fliC* (Fla^−^), **D**: PAK ∆*xcpQ* (T2SS*^−^*), **E**: PAK ∆*pscF* (T3SS*^−^*), **F**: PAK ∆*fliC*∆*xcpQ*∆*pscF* (Fla^−^ */*T2SS*^−^*/T3SS*^−^*), **G**: ultrapure flagellin deposited in culture medium, **H**: ultra-pure flagellin deposited on the upper layer of RHE. **I to** P: Immunofluorescence assays (green) using a polyclonal anti-*P. aeruginosa* antibody on RHE infected with the different PAK strains. Nuclei were counter-stained using a solution of propidium iodide red-fluorescent staining. **I**: control (sterile PBS), **J**: wild-type PAK strain, **K**: PAK ∆*fliC* (Fla^−^), **L**: PAK ∆*xcpQ* (T2SS*^−^*), **M**: PAK ∆*pscF* (T3SS*^−^*), **N**: PAK ∆*fliC*∆*xcpQ*∆*pscF* (Fla^−^ */*T2SS*^−^*/T3SS*^−^*), **O**: ultrapure flagellin deposited in culture medium, **P**: ultra-pure flagellin deposited on the upper layer of RHE.

During RHE infection with the wild-type strain (Figure 5(b)) and with T2SS or T3SS mutant strains (Figure 5(D–E)), S100A7 protein was markedly induced as compared with non-infected RHE (Figure 5(a)). In contrast, during RHE infection with ∆*fliC* (Figure 5(C)) or triple mutant strains (Figure 5(F)), S100A7 protein tissue expression was comparable to the basal level observed in a negative control. In agreement with the transcriptional results, RHE stimulated with *P. aeruginosa* flagellin deposited on the upper layer failed to induce increased production of S100A7 (Figure 5(H)) contrary to RHE stimulation with *P. aeruginosa* flagellin in culture medium (Figure 5(G)). Similar results were obtained for hBD2, although protein tissue production was weakly induced by *P. aeruginosa* infection (supplementary figure 9).

Afterwards, the invasion ability of the different bacterial strains was evaluated on the RHE model using bacterial immunostaining.

### Invasion potential of *P. aeruginosa* during infection of RHE

During RHE infection with the wild-type strain (Figure 5(J)), bacteria were found in whole tissue, including the *stratum basale*, thereby underlining the high potential of epidermis invasion by *P. aeruginosa*. In contrast, bacteria were weakly detectable in epidermis infected with *∆fliC* (Figure 5(K)) or with triple mutant (Figure 5(N)) strains, thereby suggesting a critical role of flagellum in the invasion potential of *P. aeruginosa*. At an earlier time (16 h post-infection), bacteria were detectable on the top of the epidermis infected with *∆fliC* suggesting that aflagellate bacteria can adhere to the *stratum corneum* without penetrate tissue (data not shown). The absence of functional T2SS or T3SS did not seem to influence tissue invasion by the bacterium (Figure 5(L,M)). As expected, no fluorescence was observed after RHE stimulation with *P. aeruginosa* flagellin (Figure 5(O,P)).

Finally, the cutaneous persistence ability of the wild-type and the flagellin mutant strains was evaluated using an *in vivo* mouse model of skin infection.

### Mouse skin infection

**Figure 6.**
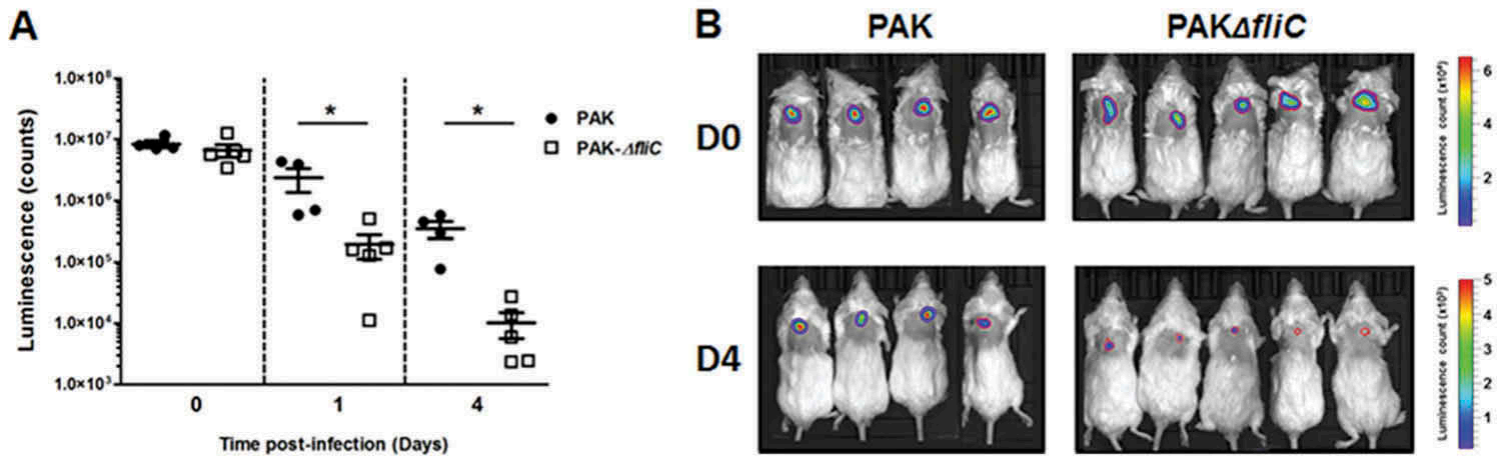
Flagellum promotes *P. aeruginosa* persistence in cutaneous infection. (A) Balb/c mice were inoculated with 1.10^7^ CFU of either PAK-*lux* (●) or PAK *∆fliC-lux* (□) strains. Infection was assessed by quantifying luminescence emission on living animals. Data represent the mean ± SEM (n = 4–5 mice/group). *p < 0.05. (B) Pictures of luminescence imaging on living animals recorded immediately after infection (D0) and after 4 days (D4) post-inoculation.

After having demonstrated the role of *P. aeruginosa* flagellum in tissue invasion, we attempted to assert its role *in vivo* using a mouse model of *P. aeruginosa* sub-cutaneous infection. At day 0, similar quantities of wild-type and *∆fliC* luminescent strains were injected subcutaneously (Figure 6A). No luminescence was noticed outside of the injection site, suggesting that none of the infected mice had systemic dissemination of the bacterium (Figure 6(B)). From the first day post-infection, a significant decrease of luminescence was observed in mice infected with the *∆fliC* strain as compared with those infected with the wild-type strain. This significant difference of luminescence was maintained after 4 days of infection, thereby suggesting a major role of flagellum in the cutaneous persistence of *P. aeruginosa*. Experiments performed on human primary keratinocytes have shown that the wild-type strain possesses a higher adhesion and intracellular invasion capacity than the *∆fliC* strain, a phenomenon which may be involved in bacterial cutaneous persistence (supplementary figure 10).

## Discussion

This study has investigated the role of major virulence factors of *P. aeruginosa* in the inflammatory response of the human epidermis. We report a strong induction of numerous pro-inflammatory cytokines, chemokines and AMPs such as CXCL1, CXCL2, CXCL5, CXCL8, CCL20, TNFα, TSLP, IL-23α, IL-32, IL-6, hBD2 and S100A7 during the infection of monolayered primary keratinocytes and reconstructed epidermis with the wild-type PAK strain. Some of these mediators have previously been described during lung [18,24,26–29] or corneal infections by *P. aeruginosa* [30], extending the potent pro-inflammatory role of this pathogen to skin infections. IL-32 is a cytokine involved in the induction of IL-6, TNFα and chemokines [31,32]. TNFα, IL-6, IL-23 and TSLP have been described as mediators involved in host defense against *P. aeruginosa* [18,26,33–35]. Together with cytokines, chemokines contribute induction and maintenance of inflammation by regulating the composition of the cellular infiltrates in the skin. Indeed, CXCL1, CXCL2, CXCL5 and CXCL8 constitute potent polymorphonuclear neutrophil attractant chemokines [36]. CCL20, which plays an important role in the homing of lymphocytes and dendritic cells to sites of inflammation, and CXCL1 and CXCL8 are upregulated at both the transcriptomic and the proteomic levels following *P. aeruginosa* infection of keratinocytes. In this way, *via* induction of pleiotropic cytokines and of a combination of chemokines, *P. aeruginosa* recruits different types of leukocytes in the skin and induces an intense pro-inflammatory response that is potentially involved in the pathophysiological process of the infection.

Some studies have demonstrated the role of the flagellin of *P. aeruginosa* in induction of IL-6 and TNFα [24,28] or CXCL8 [37,38] during mouse infections. In this work, the inflammatory response following human keratinocyte and RHE infection with flagellin-deficient strains was strongly decreased. In addition, similar pro-inflammatory profiles have been found after keratinocyte and RHE stimulation by *P. aeruginosa-*purified flagellin and whole bacterial cells. Taken together, these results demonstrate that *P. aeruginosa* flagellin, the subunit protein of flagellum, is a potent inducer of a large panel of cytokines, chemokines and AMPs in the human epidermis. This is consistent with the low percentage of acute infection induced by aflagellate mutants of *P. aeruginosa* [39], and the observation that a large proportion of isolates from human chronic infections is associated with downregulation of flagella and/or of flagella-mediated motility or are aflagellate [40].

Several AMPs are expressed in the skin such as RNase 7, S100A7, hBD2 and hBD3 [41]. Following keratinocyte and RHE infection by *P. aeruginosa*, large amounts of hBD2 and S100A7 mRNA were induced. These two AMPs play a key role in the innate immune system through their chemotactic effects and antimicrobial activities against Gram-negative bacteria including *P. aeruginosa* [42–44]. Immunohistochemistry analysis of infected RHE has shown marked production of S100A7 protein induced by the wild-type strain, which was not observed when RHE were infected by the flagellin-deficient strains. While ultrapure flagellin stimulation of primary keratinocytes resulted in enhanced expression of S100A7, RHE topical deposit of flagellin failed to induce an increase of the S100A7 tissular level, even though its addition to the RHE culture medium led to marked expression. As RHE is a waterproof multilayered structure, the absence of S100A7 induction when flagellin was added at the top of RHE suggests a need for the flagellin to be driven through the *stratum corneum* and confirms that RHE mimics the role of the physical barrier of the human skin. In agreement, it was shown that *P. aeruginosa* flagellin needs to be delivered to apical epithelial cells across the *stratum corneum* by rhamnolipids in order to induce S100A7 expression in human skin [45]. Taken as a whole, our results underline the major role of the flagellum in the induction of S100A7 expression and production during epidermis infection with *P. aeruginosa* as described previously for *E. coli* [46]. This finding also applies to other AMPs, especially hBD2. Nonetheless, during the course of infection, various mechanisms are used by Gram-negative bacteria, including *P. aeruginosa*, to sense and resist host-derived AMP-mediated killing [47].

In extracellular space, flagellin is recognized by TLR5, a plasma membrane-localized pattern recognition receptor [48] whereas in intracellular space, flagellin is sensed through NLRC4, leading to activation of the inflammasome [49]. In our work, induction of pro-inflammatory mediators is significantly decreased after keratinocyte infection with the wild-type strain in presence of the anti-TLR5 antibody. This suggests that the epidermal inflammatory response to *P. aeruginosa* infection is mainly the consequence of the recognition of the flagellin by TLR5 in extracellular space. These results are consistent with a previous study showing that TLR5 is essential to the recognition of *P. aeruginosa* flagellin both *in vivo* and *ex vivo* [38]. *P. aeruginosa* possesses other virulence factors such as LPS, which is recognized either by TLR2 or TLR4 depending on its structure [23,50]. It has been suggested that the most important system involved in the recognition of *P. aeruginosa* could be TLR and that TLR4 and TLR5 were redundant for the recognition of *P. aeruginosa* [38]. One study using an aflagellate strain in a lung infection mouse model showed that TLR4 alone was essential [51]. In contrast, using anti-TLR2 or TLR4 neutralizing antibodies, we did not observe any modulation of the inflammatory response of human keratinocytes during wild-type strain infection. This study suggests that flagellin is a key PAMP of *P. aeruginosa* during skin infection and that TLR5 is the main pattern recognition receptor involved in *P. aeruginosa* recognition by keratinocytes.

Nevertheless, *P. aeruginosa* possesses powerful secretion systems such as T2SS and T3SS contributing activation of the innate immune system and to host cell injuries. Both T2SS and the T3SS play independent roles in death due to *Pseudomonas* lung infection and are related to disease severity [18]. T3SS also enhances infection severity in several animal models: acute pneumonia [19,21,52], keratitis [52], bacteremia [22], peritonitis [53], burn infections [20] and gut-derived sepsis in neutropenia [54]. Moreover, T3SS *in vitro* detection was correlated with increased morbi-mortality and relapse in human *P. aeruginosa* ventilator-associated pneumonia and blood stream infections [55,56]. Previously, T2SS has been shown to be strongly involved in *P. aeruginosa* chronic infection [57]. In this work, the absence of functional T2SS or T3SS was not associated with significant decrease of the main inflammatory mediator expression by infected monolayered keratinocytes at an early time of infection (6 h). On the contrary, T3SS seemed to play a role at a later time of infection (16 h) given that T3SS deficient strain resulted in a decreased expression of numerous inflammatory mediators in this model. Furthermore, significantly reduced IL-6, CCL20, and hBD2 expression along with CXCL8 and CCL20 secretion by RHE were observed at 16 h post-infection using T3SS deficient strain. This finding supports a potential delayed role of T3SS in induction of the inflammatory response of keratinocytes, but the impact of this virulence factor appears minor in the early phase of *P. aeruginosa* keratinocyte infection.

The role of virulence factors in the ability of the bacterium to invade the epidermis was evaluated by immunofluorescence. After 24 h of infection, wild-type strain had spread within the RHE whereas aflagellate strains were not able to invade the epidermis. The flagellum of *P. aeruginosa* is known to be involved in bacterial motility and adhesion to host cells [58]. Our results highlight the major role of flagellum in skin invasion. On the other hand, tissue invasion of *P. aeruginosa* can be facilitated by bacterial proteases secreted by T2SS, such as elastase A, elastase B, alkaline protease and protease IV [59]. Nonetheless, T2SS and T3SS did not appear to play a major role in epidermis invasion since RHE infection with T2SS and T3SS deficient strains resulted in bacterial dissemination within tissue. Interestingly, therapies using anti-flagellin antibodies have been shown to reduce the invasiveness and mobility of *P. aeruginosa* and to increase opsono-phagocytosis in both acute pneumonia and burn models [60]. In addition, *P. aeruginosa* persistence *in vivo* after sub-cutaneous infection in mice was related to the presence of the flagellum despite its proinflammatory properties. So, the capacity of the flagellum to induce the production of AMPs in epidermis seems to be ineffective as a means of fighting *P. aeruginosa*. Finally, the higher capacity of flagellate bacteria to invade primary keratinocyte suggests that intracellular bacterial localization could be involved in cutaneous bacterial persistence.

In summary, this study provides arguments demonstrating that flagellin is the main virulence factor of *P. aeruginosa* during acute epidermis infection, inducing a huge panel of inflammatory mediators in a TLR5-dependent manner and contributing the invasiveness potential of the bacterium and skin bacterial persistence *in vivo*. Interestingly, T2SS or T3SS do not appear to be essential to the induction of a pro-inflammatory keratinocyte response to *P. aeruginosa* infection or to the ability of the bacterium to invade skin at an early time of infection.

## Materials and methods

### Isolation and culture of normal human epidermal keratinocytes from skin samples

The Ethics Committee of the Poitiers Hospital approved the use of human skin samples for research studies. After the provision of fully informed consent, normal abdominal or breast skin were obtained from patients undergoing plastic surgery. Small pieces of skin were thoroughly washed with phosphate-buffered saline solution free of calcium and magnesium (PBS; Gibco) after removal of fat. The skin was minced into fragments of about 125 mm^2^ using scalpel blades. Skin samples were incubated overnight at 4°C in a dispase solution (25 U/mL; Life Technologies). Epidermal sheets were removed from the dermis, and keratinocytes were dissociated by trypsin digestion (trypsin-EDTA; Gibco) for 15 min at 37°C. The cell suspension was then filtered through a 280-µm sterile filter. Dulbecco’s modified essential medium (DMEM; Gibco) supplemented with 10% of fetal bovine serum (FBS; Gibco) was added vol/vol and the suspension was centrifuged at 1500 rpm for 10 min. Keratinocytes were seeded at a density of 10^7^ cells in 75-cm^2^ tissue culture flask in Keratinocyte-Serum Free Medium (K-SFM) supplemented with bovine pituitary extract (25 μg/mL) and recombinant epidermal growth factor (0.25 ng/mL; all were purchased from Invitrogen Life Technologies). The cultures were incubated at 37°C in a humidified atmosphere with 5% CO_2_ until confluency and then stored frozen in nitrogen until use. Finally, keratinocytes were seeded in sterile 24-well culture plates at a density of 4 × 10^4^ cells/well in K-SFM supplemented with bovine pituitary extract and epidermal growth factor and cultured to 80% confluence. Cells were then starved overnight in K-SFM alone before stimulation.

### RHE culture

RHE was prepared as previously described [61]. Suspensions of cultured primary human keratinocytes (see above) were further cultured on polycarbonate culture inserts (Millipore) in Epilife medium (Thermo Fisher Scientific) supplemented with 1.5 mM calcium chloride and 50 µg/mL ascorbic acid, and then transferred to the air–liquid interface for 10 days.

### Bacterial strains, infection and stimulation protocols

The bacterial strains and plasmids used in this study are listed in Table 1. *Pseudomonas aeruginosa* and *Escherichia coli* strains were grown in Luria-Bertani (LB) broth at 37°C. Gentamicin was used at final concentrations of 30 µg/mL for *P. aeruginosa*, and 15 µg/mL for *E. coli*. Plasmids pTNS3 and pUC18T-miniTn7T-*lux* were co-introduced into *P. aeruginosa* strains by electroporation. Clones with transposon insertion were selected on LB-agar plates supplemented with gentamicin and chromosomal integration was verified by PCR as described in [62]. The *P. aeruginosa* wild type strain PAK used in this study is a common strain fully expressing virulence factors including T2SS, T3SS, pili and flagellum [26]. PAK ∆*fliC*, a flagellin-deficient strain (Fla-), is derivated from the wild type strain where *fliC* has been deleted [63]. In a same way, PAK ∆*pscF* (a T3SS deficient strain), PAK *∆xcpQ* (a T2SS-deficient strain), and PAK *∆fliC∆xcpQ∆pscF* (deficient for Fla, T2SS and T3SS) were generated [64]. These strains were cultured on Mueller-Hinton (MH; Oxoid) agar plates incubated for 24 h at 37°C in aerobic atmosphere. For cell infection assays, bacterial suspensions of each strain were prepared in the cell culture medium using 24-h bacterial cultures. Bacterial concentration was determined by measuring the optical density (OD) of the suspension at 600 nm and by colony forming unit (CFU) counts for each strain controlled by serial 10-fold dilutions of the bacterial suspensions and plating on MH agar plates.

**Table 1.** Strains and plasmids used in this study.

Strains and plasmids	Characteristics	Source or reference
***P. aeruginosa* strains**		
**PAK**	*P. aeruginosa* strain K, wild-type	[25]
**PAK *∆xcpQ***	In-frame partial deletion of *xcpQ* in strain PAK: defective for type II secretion system (T2SS^−^)	[11]
**PAK *∆pscF***	In-frame partial deletion of *pscF* in strain PAK: defective for type III secretion system (T3SS^−^)	[11]
**PAK *∆fliC***	In-frame partial deletion of *fliC* in strain PAK; non-motile (Fla^−^)	[11]
**PAK *∆fliC∆xcpQ∆pscF***	In-frame partial deletion of *xcpQ* and *pscF* in strain PAK*∆fliC*: defective for both type II (T2SS^−^) and type III secretion system (T3SS^−^); non-motile (Fla^−^)	[11]
**PAK-*lux***	PAK strain in which *luxCDABE* gene expression cassette from plasmid pUC18T-miniTn7T-*lux* was inserted into the chromosomal *att* site	This work
**PAK *∆fliC-lux***	PAK-*∆fliC* strain in which *luxCDABE* gene expression cassette from plasmid pUC18T-miniTn7T-*lux* was inserted into the chromosomal *att* site	This work
**Plasmids^a^**		
**pUC18T-miniTn7T-*lux***	KC848884; suicide vector for shuttling single copies of genes directly to the chromosome via a mini-Tn*7* element; *aacC1* gene encoding gentamicin resistance marker on Tn*7* element; contains *oriT* for mobilization; P1 integron promoter driven expression of *luxCDABE*; Amp^r^ Gm^r^	[65]
**pTNS3**	Helper plasmid encoding the Tn*7* site-specific transposition pathway; Amp^r^	[62]

^a^Amp^r^ and Gm^r^ are resistant to ampicillin and gentamycin, respectively.

### Infection and stimulation protocols

Bacteria were grown on MH plates at 37°C overnight and resuspended in sterile PBS to an OD_660nm_ of 0.1 corresponding to a bacterial concentration of 10^8^ CFU/mL. Keratinocytes were infected at a multiplicity of infection (MOI) of 1 and incubated for 6 h and 16 h at 37°C in 5% CO_2_. TLR signaling was investigated with neutralizing monoclonal antibodies (Abs) against human TLR2, TLR4 and TLR5 (TLR2 IgA2, TLR4 IgG1 and TLR5 IgA2, 5 µg/mL; InvivoGen) and corresponding isotype control Abs (human IgA2, 5 µg/mL (Invivogen) and mouse IgG1, 5 µg/mL (R&D systems)). Keratinocytes were pre-treated for 2 h by different Abs before being infected by *P. aeruginosa*. Keratinocytes were also stimulated with 1 µg/mL of ultrapure *P. aeruginosa* flagellin (InvivoGen). For RHE infection experiments, 2 x 10^3^ bacteria were deposited on the upper layer of RHE for 4 h at 37°C in 5% CO_2_. Bacterial suspensions were then removed and RHE were incubated for an additional period of 12 or 20 h. Cell culture supernatants were stored at −20°C until ELISA assays. In addition, RHE were stimulated with 1 µg/mL of ultrapure *P. aeruginosa* flagellin added to the medium culture or with 200 µl of ultrapure *P. aeruginosa* flagellin at a final concentration of 1 µg/mL diluted in PBS deposited on the upper layer of RHE. PBS alone was used as control.

### RNA extraction, reverse transcription and real-time PCR analysis

Total RNA extraction from keratinocytes was performed using the Nucleo-Spin XS RNA extraction kit according to the manufacturer’s instructions (Macherey-Nagel). Total RNA from RHE was extracted using TriPure Isolation Reagent following the manufacturer’s recommendations (Roche). RNA concentrations and purity were determined using the Nanodrop 2000 spectrophotometer (Thermo Fisher Scientific). Total RNA (1 µg) was reverse transcribed using SuperScript II kit (Invitrogen). Quantitative real time (RT)-PCR was performed in 96-well plates using LightCycler-FastStart DNA Master^Plus^SYBR GREEN I kit (Roche) on LightCycler 480 (Roche).

Reaction mixtures consisted of 1X DNA Master Mix (Applied Biosystems), 1 μM forward and reverse primers designed using Primer 3 software and 12.5 ng of cDNA template in a total volume of 10 µl. PCR conditions were as follows: 5 min at 95°C, 40 amplification cycles comprising 20 s at 95°C, 15 s at 64°C and 20 s at 72°C. Samples were normalized with regard to two independent control housekeeping genes (Glyceraldehyde-Phospho-Dehydrogenase and 28S rRNA gene) and reported according to the ΔΔCT method as RNA fold increase: 2^ΔΔCT^ = 2^ΔCT sample – ΔCT reference^.

### Enzyme-linked immunosorbant assay (ELISA)

Levels of CXCL1, CXCL8 and CCL20 in cell culture supernatants were determined in duplicates for each sample using human ELISA kits (R&D systems for CXCL1 and CCL20; PeproTech for CXCL8) in accordance with the manufacturers’ specifications.

### Immunostaining

Immunostaining was performed on RHE infected by different *P. aeruginosa* strains or stimulated by ultra-pure *P. aeruginosa* flagellin for 24 h. RHE were washed and fixed with formaldehyde solution before dehydratation with increasing ethanol concentrations and embedding in paraffin. Sections were carried out using a microtome (4 mm thickness). Deparaffinised sections were washed, incubated with hydrogen peroxide and then incubated with anti-hBD2 (SantaCruz, SC-20,798) or anti-S100A7 (Novus NB100-56,559) antibodies. Finally, sections were detected using a biotin-conjugated secondary antibody (*Novolink* Polymer Detection System, Leica, RE7140-K). After peroxidase-conjugated streptavidine (*Novolink* Polymer Detection System) and peroxidase substrate addition (Dako, Substrate hypersensible AEC+), nuclei were counter-stained using a hematoxylin solution. Deparaffinised sections washed for immunofluoresecence staining were incubated 20 minutes in citric acid buffer (pH = 6) successively at 92°C and ambient room temperature. Sections were then blocked with a PBS-BSA 1% solution, incubated with a rabbit polyclonal anti-*P. aeruginosa* antibody (Abcam) for 2 h, and detected using a GAR-Alexa 488 antibody (Molecular probes). Nuclei were counter-stained using a solution of propidium iodide red-fluorescent staining (Sigma). Finally, sections were mounted in a fluorescent mounting medium. Sections were observed using a NIKON E400 microscope. The images were captured using a NIKON DS-Ri1 and processed with NISElements 3.10 software.

### Mouse infection by *P. aeruginosa* and luminescence measurements

Balb/c mice (males) were used at ∼8 weeks of age and supplied by Janvier Laboratories. Animals were maintained according to the guidelines issued by the *Comité d’Ethique en Expérimentation Animale Val de Loire* and the European Union for the care and use of animals in research protocols. All mice were housed under a reverse light–dark cycle, under standard conditions, with food and water available *ad libitum*. Mice were anesthetized by intraperitoneal injection of a mixture of ketamine-xylazine. For infection experiments, *P. aeruginosa* was grown overnight in LB broth and further transferred into fresh medium and grown for 4–5 h to mid-log phase. The bacteria cultures were centrifuged at 4000 × *g* for 15 min and the cell pellets washed twice with PBS. The bacterial pellet was diluted in its original volume and the OD adjusted to yield the approximate desired inoculum of 1 x 10^7^ CFU. A 100-μl bacterial suspension was administrated by sub-cutaneous injection on back mice. The inocula were verified by serial 10-fold dilutions of the bacterial suspensions and plated on LB agar plates.

Photon emission of luminescent *P. aeruginosa* (wild-type and *∆fliC* strains) in the mouse skin was measured using the IVIS Lumina XR system (Perkin Elmer), which includes an IVIS charge-coupled device camera coupled with the Living Image software package (Perkin Elmer). Analysis of photons was done under isoflurane inhalation anesthesia. A digital false-color photon emission image of the mouse was generated, and photons were counted using a 3-minute acquisition time with the following settings: Medium binning (M), Field Of View: 12.5 (D), f1. Image analysis and luminescence quantification were performed with Living Image software. Region Of Interest (ROI) were drawn automatically using the ‘autoROI’ function of the software (threshold 6%).

### Statistical analysis

Results were analysed by GraphPad Prism version 5. The statistical significance of the difference between two groups was evaluated by the one-way ANOVA test followed by the Dunnett’s test and by t-test. For animal experiments the statistical significance of the difference between the two groups was evaluated by the two-tailed non-parametric Mann Whitney test. Differences were considered to be significant at *p *< 0.05.

## List of abbreviations and acronyms

Abs: antibodies
AMP: antimicrobial peptide
CFU: colony forming unit
ELISA: enzyme-linked immunosorbent *assay*
hBD2: human beta-defensin 2
IL: interleukin
K-SFM: keratinocyte-serum free medium
LB: Luria-Bertani
LPS: lipopolysaccharide
MH: Mueller-Hinton
MOI: multiplicity of infection
OD: optical density
PAMPs: pathogen-associated molecular patterns
*P.*: *aeruginosa: Pseudomonas aeruginosa*
PBS: phosphate-buffered saline
RHE: reconstructed human epithelium
RNAse 7: ribonuclease 7
ROI: region of interest
RT-PCR: real time polymerase chain reaction
S100A7: S100 calcium-binding protein A7
SFM: serum-free medium
T2SS: type 2 secretion system
T3SS: type 3 secretion system
TLR: toll-like receptor
TNFα: tumor necrosis factor α
TSLP: thymic stromal lymphopoietin
