# Supplementary material for: Pseudomonas aeruginosa flagellum is critical for invasion, cutaneous persistence and induction of inflammatory response of skin epidermis
Source: Virulence. 2018 Aug 2;9(1):1163–75. doi: 10.1080/21505594.2018.1480830 (PMC6086312; doi:10.1080/21505594.2018.1480830)
Supplement: Supplemental Material [file kvir-09-01-1480830-s001.pdf]

SUPPLEMENTARY MATERIAL

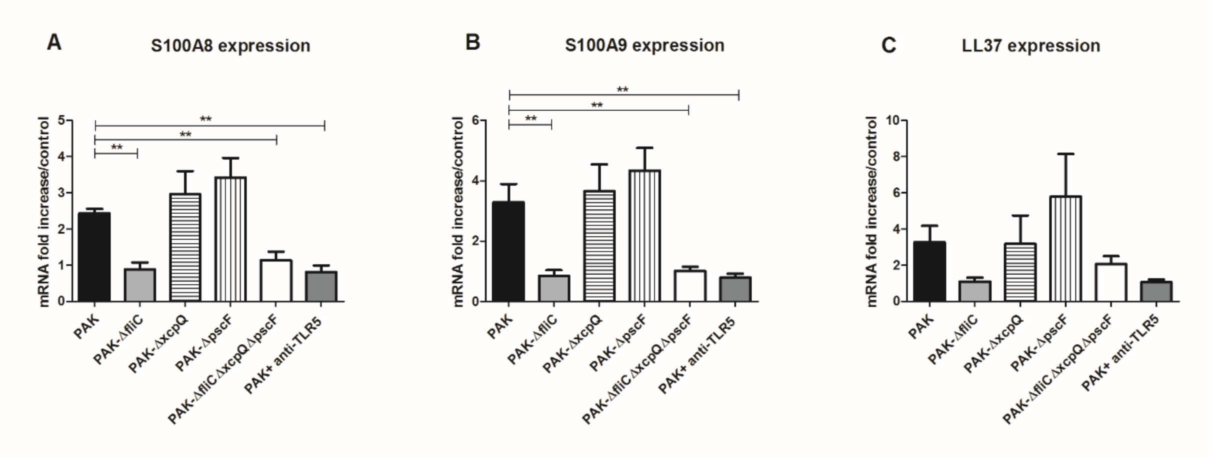

<u>Supplementary figure 1.</u> AMP mRNA expression in response to keratinocyte infection by each kind of bacterial strain. S100A8 (A), S100A9 (B), LL 37 (C) mRNA expression by keratinocytes infected for 6 h with wild-type PAK, PAK Δ*fliC* (Fla^-), PAKΔ*xcpQ* ($T2SS^-$), PAKΔ*pscF* ($T3SS^-$), PAK Δ*fliC*Δ*xcpQ*Δ*pscF* (Fla^-/$T2SS^-$/$T3SS^-$) strains or wild-type PAK strain in presence of the anti-TLR5 monoclonal antibody. mRNA expression levels are expressed as the fold increase above unstimulated cultures. Data are represented as mean + SEM of five independent experiments. ***$p<0.01$.*

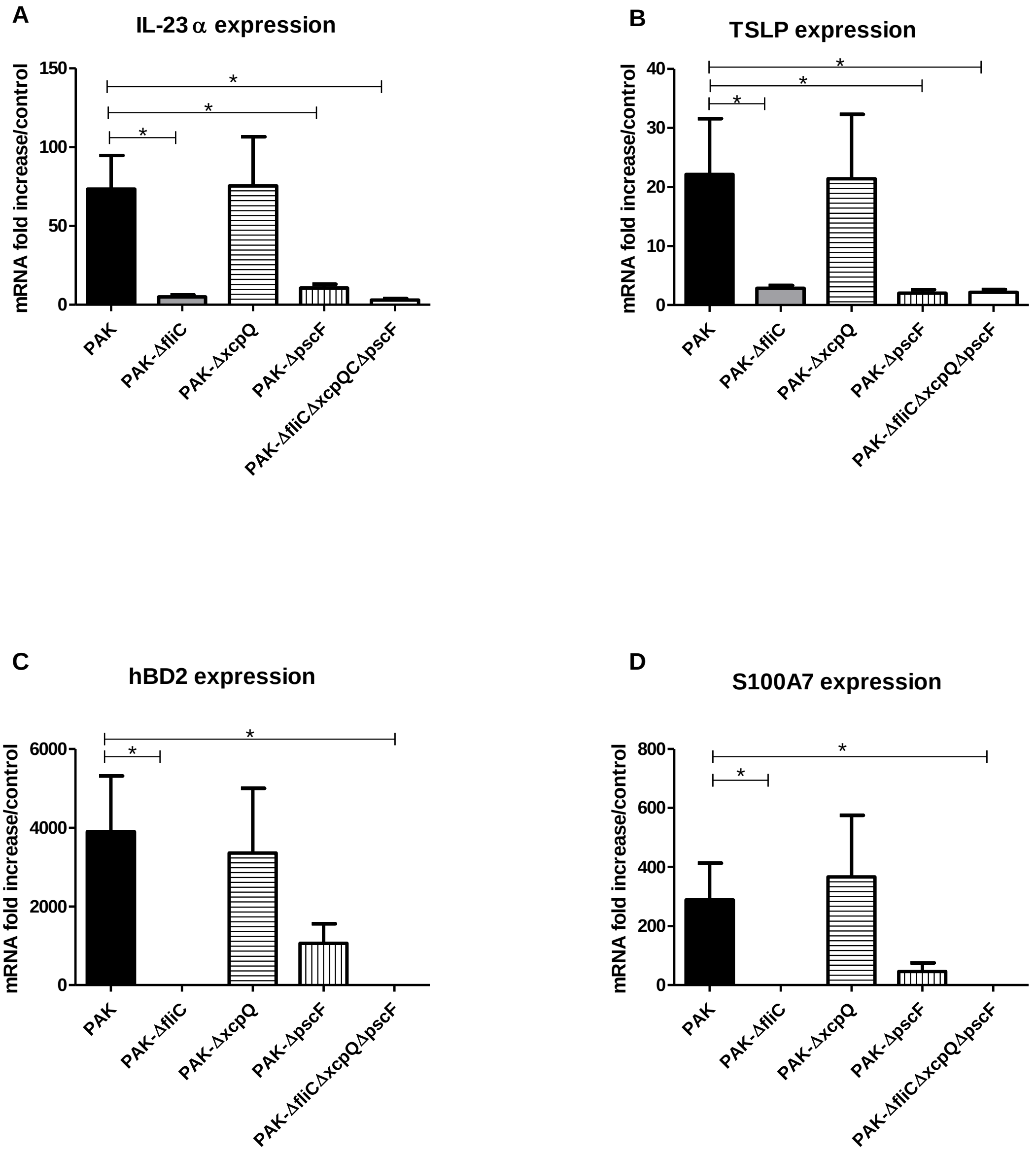

<u>Supplementary figure 2.</u> **Cytokine and AMP mRNA expression in response to keratinocyte infection by each kind of bacterial strain.** IL-23α (A), TSLP (B), hBD2 (C) and S100A7(D) mRNA expression by keratinocytes infected for 16 h with wild-type PAK, PAK Δ*fliC* (Fla$^-$), PAKΔ*xcpQ* (T2SS$^-$), PAKΔ*pscF* (T3SS$^-$), PAK Δ*fliC*Δ*xcpQ*Δ*pscF* (Fla$^-$/T2SS$^-$/T3SS$^-$) strains. mRNA expression levels are expressed as the fold increase above unstimulated cultures. Data are represented as mean + SEM of five independent experiments. **p< 0.05.*

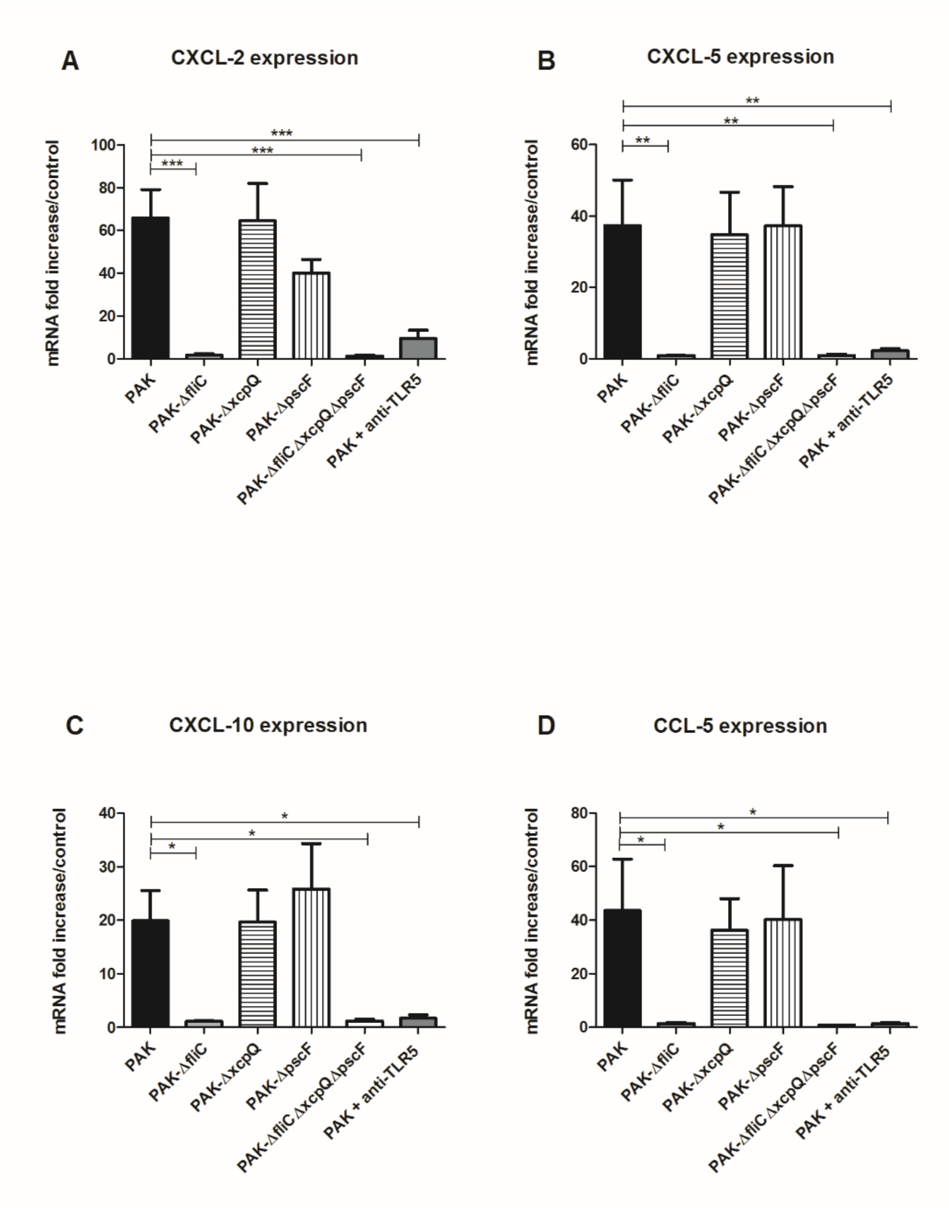

<u>Supplementary figure 3.</u> Chemokine mRNA expression in response to keratinocyte infection by each kind of bacterial strain. CXCL2 (A), CXCL5 (B), CXCL10 (C), CCL5 (D) mRNA expression by keratinocytes infected for 6 h with wild-type PAK, PAK Δ*fliC* (Fla$^-$), PAK Δ*xcpQ* (T2SS$^-$), PAK Δ*pscF* (T3SS$^-$), PAK Δ*fliC*Δ*xcpQ*Δ*pscF* (Fla$^-$/T2SS$^-$/T3SS$^-$) strains or wild-type PAK strain in presence of the anti-TLR5 monoclonal antibody. mRNA expression levels are expressed as the fold increase above unstimulated cultures. Data are represented as mean + SEM of five independent experiments. **p< 0.05, **p<0.01* and **** p<0.001.*

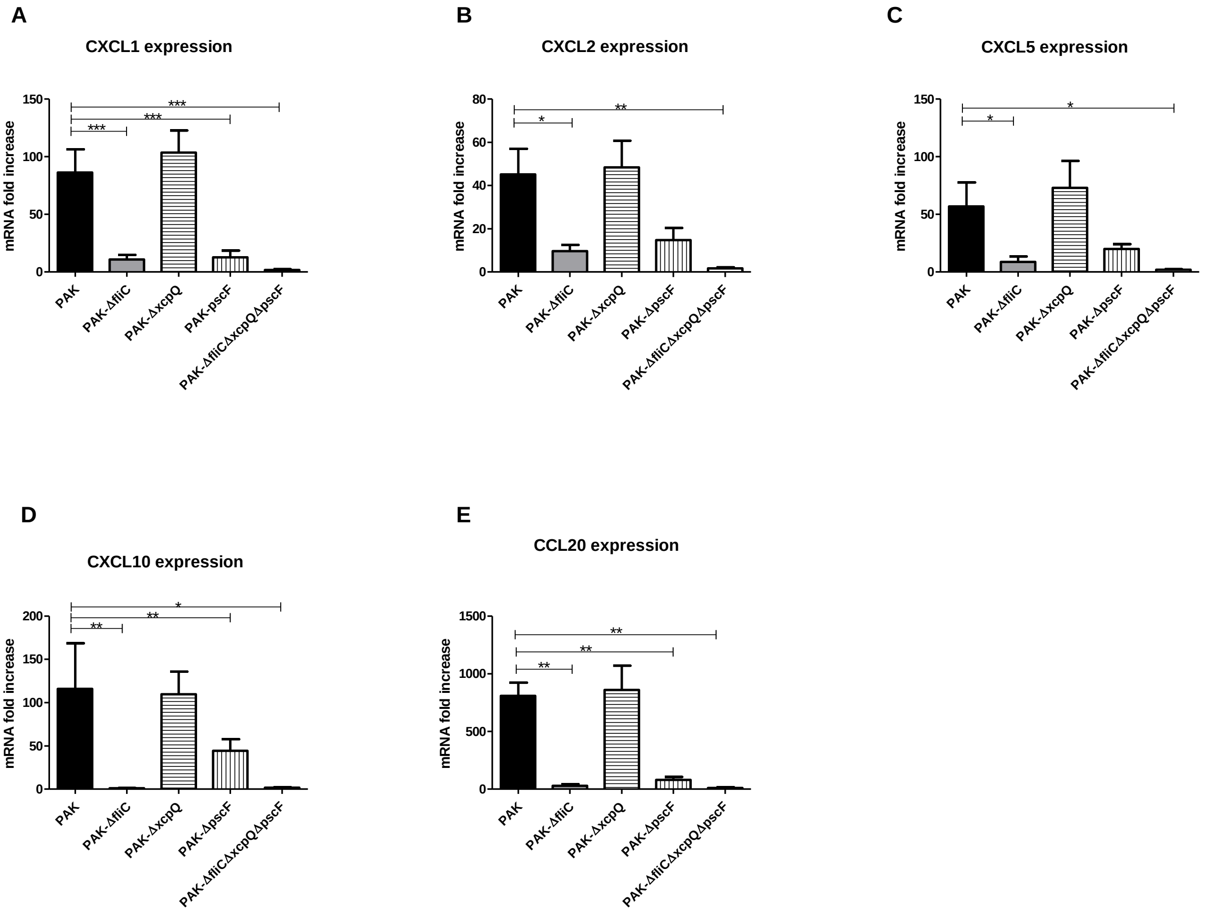

<u>Supplementary figure 4.</u> Chemokine mRNA expression in response to keratinocyte infection by each kind of bacterial strain. CXCL1 (A), CXCL2 (B), CXCL5 (C), CXCL10 (D), CCL20 (E) mRNA expression by keratinocytes infected for 16 h with wild-type PAK, PAK Δ*fliC* (Fla$^-$), PAKΔ*xcpQ* (T2SS$^-$), PAKΔ*pscF* (T3SS$^-$), PAK Δ*fliC*Δ*xcpQ*Δ*pscF* (Fla$^-$/T2SS$^-$/T3SS$^-$) strains. mRNA expression levels are expressed as the fold increase above unstimulated cultures. Data are represented as mean + SEM of five independent experiments. **p< 0.05, **p<0.01* and **** p<0.001.*

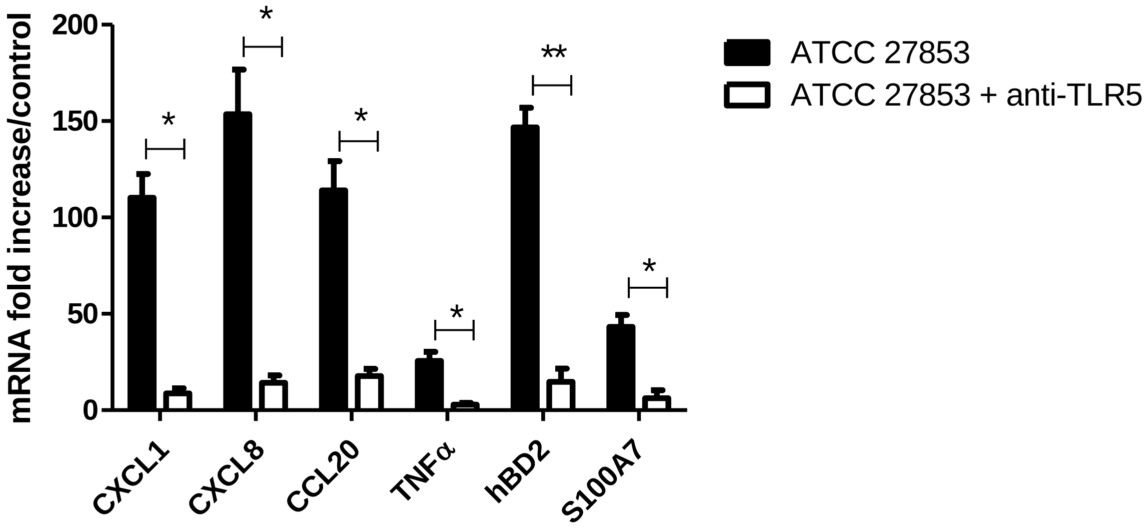

<u>Supplementary figure 5.</u> **Inflammatory mediator mRNA expression in response to keratinocyte infection by *P. aeruginosa* ATCC 27853 strain in presence or absence of TLR5 blocking antibody.** CXCL1, CXCL8, CCL20, TNFα, hBD2, S100A7 mRNA expression by keratinocytes infected for 6 h with ATCC 27853 strain in presence or absence of the anti-TLR5 monoclonal antibody. mRNA expression levels are expressed as the fold increase above unstimulated cultures. Data are represented as mean + SEM of three independent experiments. **p< 0.05* and ***p<0.01.*

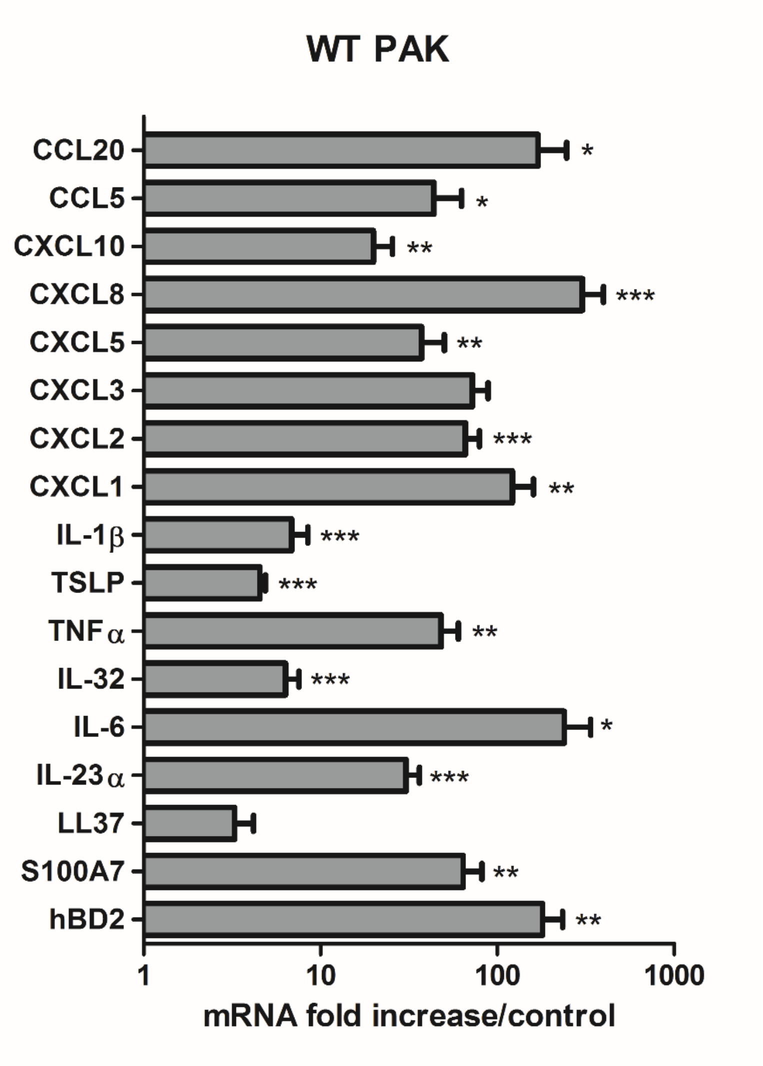

__Supplementary figure 6.__ **Inflammatory mediator mRNA expression in response to keratinocyte infection by wild-type *P. aeruginosa* PAK strain (WT PAK).** CCL20, CCL5, CXCL10, CXCL8, CXCL5, CXCL3, CXCL2, CXCL1, IL-1β, TSLP, TNFα, IL-32, IL-6, IL-23α, hBD2, S100A7 mRNA expression by keratinocytes infected for 6 h with WT PAK strain. mRNA expression levels are expressed as the fold increase above unstimulated cultures. Data are represented as mean + SEM of five independent experiments. *$p < 0.05$, **$p<0.01$ and *** $p<0.001$.

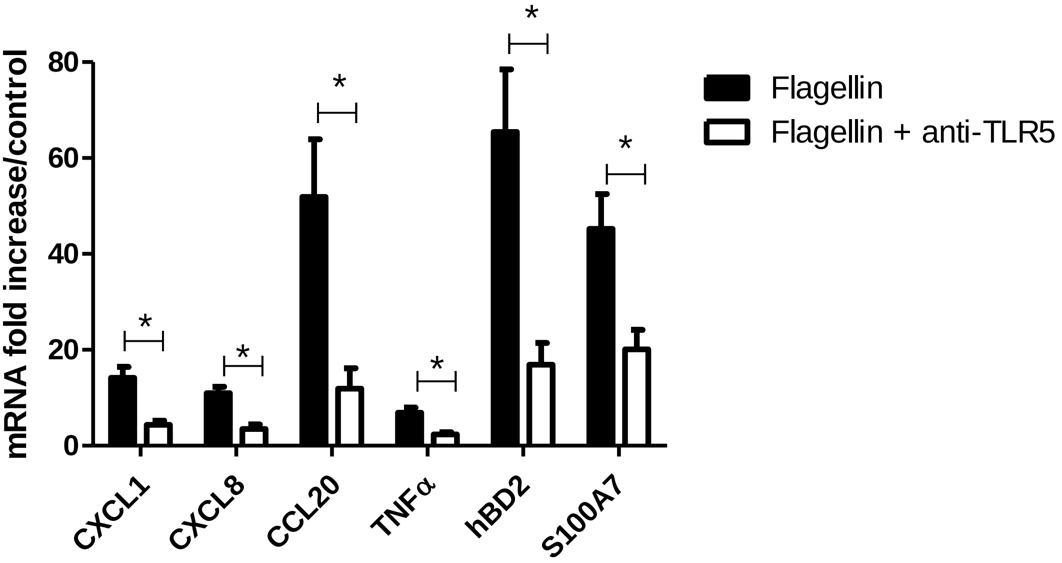

<u>Supplementary figure 7.</u> Inflammatory mediator mRNA expression in response to keratinocyte stimulation by *P. aeruginosa* flagellin in presence or absence of TLR5 blocking antibody. CXCL1, CXCL8, CCL20, TNFα, hBD2, S100A7 mRNA expression by keratinocytes stimulated for 6 h with ultra-pure flagellin from *P. aeruginosa* (1 µg/ml) in presence or absence of an anti-TLR5 monoclonal antibody. Data are represented as mean + SEM of four independent experiments. $*p < 0.05$.

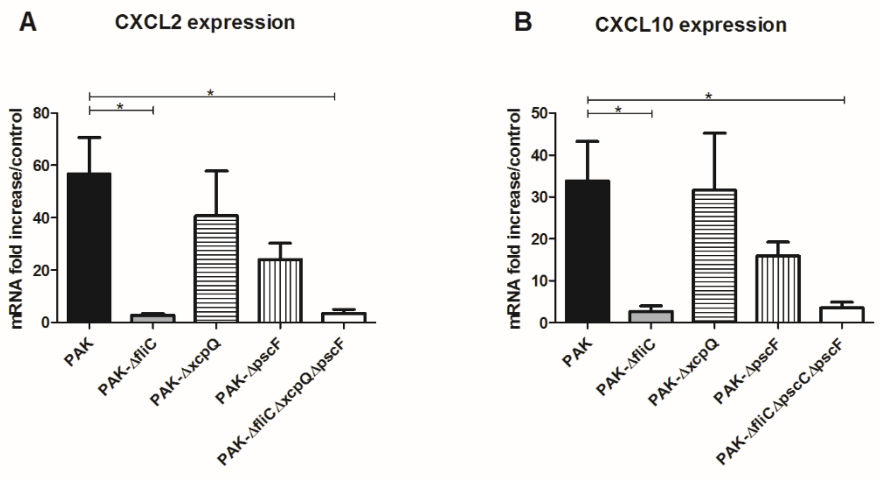

<u>Supplementary figure 8.</u> Chemokine mRNA expression in response to RHE infection by each kind of bacterial strain. CXCL2 (A) and CXCL10 (B) mRNA expression by RHE infected with wild-type PAK, PAK Δ*fliC* (Fla$^-$), PAK Δ*xcpQ* (T2SS$^-$), PAK Δ*pscF* (T3SS$^-$) or PAK Δ*fliC*Δ*xcpQ*Δ*pscF* (Fla$^-$/T2SS$^-$/T3SS$^-$) strains. mRNA expression levels are expressed as the fold increase above unstimulated cultures. Data are represented as mean + SEM of five independent experiments. *$*p< 0.05$.*

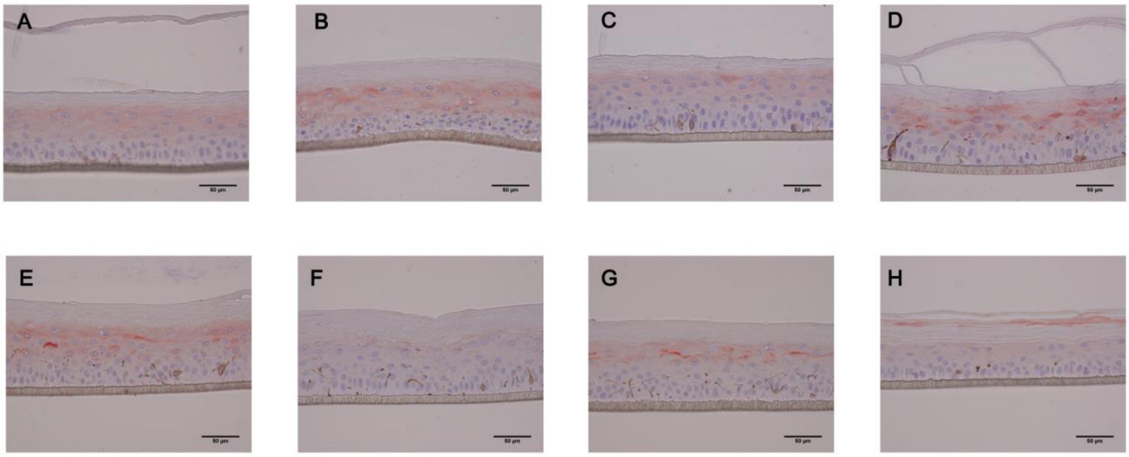

<u>**Supplementary figure 9.**</u> **A to H: Level of hBD2 tissue production assessed by immunohistochemistry on RHE infected with the different PAK strains of *P. aeruginosa* for 24 h**. **A:** control (PBS sterile), **B:** wild-type PAK strain, **C:** PAK Δ*fliC* (Fla$^-$), **D:** PAK Δ*xcpQ* (T2SS$^-$), **E:** PAK Δ*pscF* (T3SS$^-$), **F:** PAK Δ*fliC*Δ*xcpQ*Δ*pscF* (Fla$^-$/T2SS$^-$/T3SS$^-$), **G:** ultrapure flagellin deposited in culture medium, **H:** ultra-pure flagellin deposited on the upper layer of RHE.

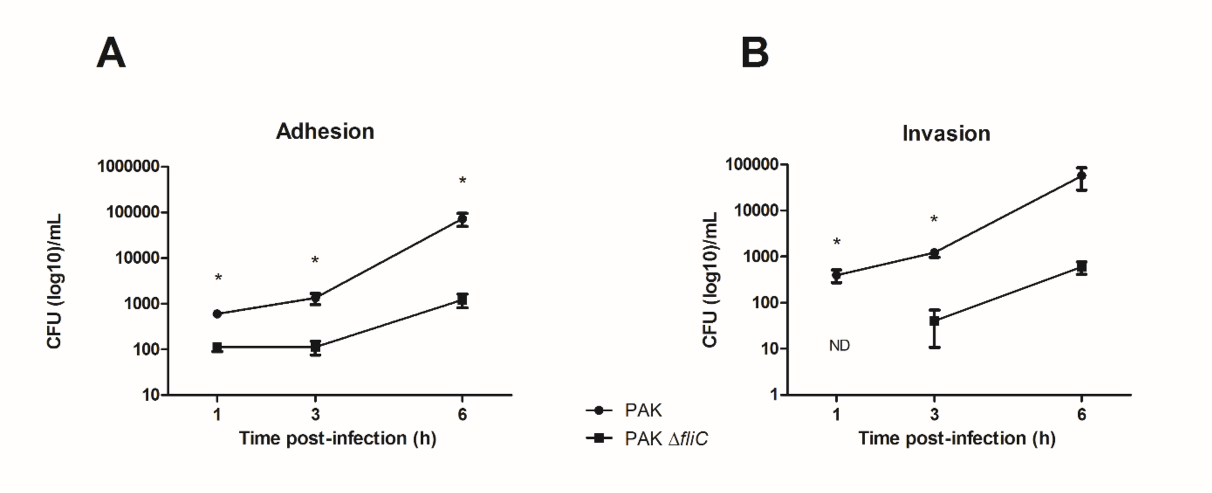

<u>Supplementary figure 10.</u> Keratinocyte adhesion (A) and invasion (B) by wild-type PAK strain (PAK) and *ΔfliC* (PAK *ΔfliC*) strains after 1, 3 or 6 hours of infection. Keratinocytes were infected with the wild-type PAK and the PAK *ΔfliC* (Fla-) strains at a multiplicity of infection of 1 and incubated for 1, 3 or 6 h at 37 °C in 5 % CO_2. For adhesion experiments, keratinocytes were washed three times with sterile PBS before being lysed with 0.25 % Triton X-100 (15 min). For invasion experiments, keratinocytes were washed three times with sterile PBS before being treated with K-SFM medium containing gentamicin (200 µg/mL) for 1 h at 37 °C in 5 % CO_2. Then, keratinocytes were washed three times with sterile PBS before being lysed with 0.25 % Triton X-100 (15 min). Finally, CFU counts for each condition were controlled by serial 10-fold dilutions of the cell lysates and plating on MH agar plates. Data are represented as mean ± SEM of three independent experiments performed in duplicate. *$p < 0.05$. ND: not detected.
